# Anti-pentraxin 3 auto-antibodies might be protective in lupus nephritis: a large cohort study

**DOI:** 10.1080/0886022X.2017.1308258

**Published:** 2017-04-10

**Authors:** Mo Yuan, Ying Tan, Yun Pang, Yong-zhe Li, Yan Song, Feng Yu, Ming-hui Zhao

**Affiliations:** a Key laboratory of Renal Disease, Ministry of Health of China, Key Laboratory of CKD Prevention and Treatment, Ministry of Education of China, Renal Division, Department of Medicine, Peking University First Hospital; Institute of Nephrology, Peking University, Beijing, PR China;; b Department of Rheumatology and Clinical Immunology, Peking Union Medical College Hospital; Chinese Academy of Medical Sciences & Peking Union Medical College; Key Laboratory of Rheumatology and Clinical Immunology, Ministry of Education, Beijing, PR China;; c Department of Nephrology, the First Affiliated Hospital of Chinese PLA General Hospital, Beijing, PR China;; d Peking-Tsinghua Center for Life Sciences, Beijing, PR China

**Keywords:** Systemic lupus erythematosus, lupus nephritis, anti-pentraxin 3 auto-antibodies, pentraxin 3, renal damage

## Abstract

**Objectives:** Anti-pentraxin 3 (PTX3) auto-antibodies were found to be associated with the absence of renal involvement in systemic lupus erythematosus (SLE). This study is to investigate the prevalence of anti-PTX3 auto-antibodies and their clinical significance based on a large Chinese lupus nephritis cohort.

**Methods:** One hundred and ninety-six active lupus nephritis patients, 150 SLE patients without clinical renal involvement, and 100 healthy controls were enrolled. Serum anti-PTX3 auto-antibodies and PTX3 levels were screened by enzyme-linked immunosorbent assay (ELISA). The associations between anti-PTX3 auto-antibodies and clinicopathological parameters in lupus nephritis were further analyzed.

**Results:** Anti-PTX3 auto-antibodies were less prevalent in active lupus nephritis patients compared with SLE without renal involvement (19.4% (38/196) versus 40.7% (61/150), *p* < .001). The serum levels of anti-PTX3 auto-antibodies were negatively correlated with proteinuria in lupus nephritis (*r* = −.143, *p* = .047). The levels of proteinuria, serum creatinine, and the prevalence of thrombotic microangiopathy were significantly higher in patients with higher PTX3 levels (≥3.207 ng/ml) and without anti-PTX3 auto-antibodies compared with patients with lower PTX3 levels (<3.207 ng/ml) and with anti-PTX3 auto-antibodies (4.79 (3.39–8.28) versus 3.95 (1.78–7.0), *p* = .03; 168.84 ± 153.63 versus 101.44 ± 47.36, *p* = .01; 34.1% (14/41) versus 0% (0/9), *p* = .04; respectively).

**Conclusion:** Anti-PTX3 auto-antibodies were less prevalent in active lupus nephritis patients compared with SLE without renal involvement and associated with less severe renal damage, especially with the combined evaluation of serum PTX3 levels.

## Introduction

Lupus nephritis is one of the most frequent and severe manifestations of systemic lupus erythematosus (SLE)^1^ and the pathogenesis has not been clarified yet. It is generally accepted that lupus nephritis was involved in the disturbed apoptosis and impaired clearance, which might contribute to the rupture of tolerance against auto-antigens and the generation of auto-antibodies.^2–4^ Thus, abundant deposition of preformed immune complexes might incite immune inflammatory renal damage through recruitment of inflammatory cells and complement activation.^5^^,^^6^

Recent studies suggest that once the inflammatory process is established, the balance between pro-inflammatory and anti-inflammatory auto-antibodies may influence propagation or attenuation renal inflammation in lupus nephritis. Several types of auto-antibodies are thought to play an important role in the development and progression of lupus nephritis, such as anti-double-stranded DNA (ds-DNA) antibodies, anti-nucleosome antibodies, anti-C1q antibodies,^7^^,^^8^ auto-antibodies against podocyte antigens,^9^^,^^10^ etc, while hindrance of inflammatory tissue damage could also be mediated by protective auto-antibodies, such as anti-dsDNA IgM.^11^

Pentraxin 3 (PTX3) belongs to the long-pentraxin family and is a multifactorial acute-phase glycoprotein with a highly conserved primary structure.^12^^,^^13^ It is rapidly secreted at inflammatory sites by immune and non-immune cells, including dendritic cells, macrophages, fibroblasts, activated endothelial cells, mesangial cells, and tubular epithelial cells in response to pro-inflammatory factors.^14^^,^^15^ Our previous study demonstrated that circulating levels and renal deposition of PTX3 were increased in active lupus nephritis patients, which suggested that PTX3 might be involved in the pathogenesis of lupus nephritis.^16^

Interestingly, recent studies demonstrated the different prevalence of anti-PTX3 auto-antibodies in SLE patients.^17^^,^^18^ Experimental animal model further supported that the anti-PTX3 auto-antibodies might exert a protective role against renal immune inflammatory damage,^19^ although the detailed descriptions between anti-PTX3 auto-antibodies and its clinical significance in human lupus nephritis was lacked.

Herein, we detected the prevalence of anti-PTX3 auto-antibodies in a large lupus nephritis cohort and further analyzed the associations between them and clinicopathological features.

## Materials and methods

### Patients and serum

The study group included 196 patients with renal biopsy-proven lupus nephritis, diagnosed between January 2000 and August 2010 in Peking University First Hospital. 150 SLE patients with negative urinalysis (urinary protein excretion <0.5 g/d or <3 + and without any cast, e.g. red cell cast, hemoglobin cast, granular cast or mixed cast) and normal renal function (non-renal SLE group) from two centers, including 75 from Peking University First Hospital and 75 from Peking Union Medical College Hospital, were used as disease control. All the patients fulfilled the 1997 American College of Rheumatology (ACR) revised criteria for SLE.^20^

Serum samples from all the patients were collected on the day of renal biopsy or at presentation before immunosuppressive treatment in patients at active phase. Serum from 100 healthy volunteers, matched for age and gender, were selected as normal controls.

All serum samples were stored in aliquots at −80 °C until use. Avoid repeated freeze–thaw cycles. Informed consent was obtained for blood sampling and renal biopsy from each patient. The research was in compliance with the Declaration of Helsinki and approved by the local ethical committees.

### Clinical evaluation

The following clinical data were gathered and analyzed: gender, age, fever, eruption, photosensitivity, oral ulcer, alopecia, arthralgia, serositis, neurologic disorder, anemia, thrombocytopenia, leukocytopenia, hematuria, leukocyturia, and acute renal failure. The clinical disease activity was measured using the Systemic Lupus Erythematosus Disease Activity Index (SLEDAI).^21^^,^^22^ The patients were followed up in outpatient lupus clinics. The primary endpoint was defined as death, and the secondary endpoints were defined as end-stage renal disease (ESRD) or doubling of serum creatinine levels.

### Laboratory assessment

Serum antinuclear antibodies (ANA) and anti-double-stranded DNA antibodies were detected using the indirect immunofluorescence assay (EUROIMMUN, Lübeck, Germany). Anti-cardiolipin antibodies were detected using enzyme-linked immunosorbent assay (ELISA) (EUROIMMUN). Anti-ENA antibodies were detected using an immunodotting assay (EUROIMMUN). Serum C3 was determined using the rate nephelometry assay (IMMAGE; Beckman-Coulter, Brea, CA; normal range >0.85 g/l).

### Renal histopathology

The renal biopsy specimens were examined by light microscopy, direct immunofluorescence, and electron microscopy techniques.

### Light microscopy examination

Renal biopsy specimens were fixed in 4.5% buffered formaldehyde for light microscopy. Consecutive serial 3 μm sections were used for histological staining. Stains employed included hematoxylin and eosin (H&E), periodic acid-Schiff (PAS), silver methenamine (Meth), and Masson trichrome.

Lupus nephritis was classified according to the International Society of Nephrology/Renal Pathology Society (ISN/RPS) 2003 classification system.^23^ Pathological parameters such as activity indices and chronicity indices were determined by two independent experienced pathologists using a previously reported system involving semi-quantitative scoring of specific biopsy features with minor modification.^24^^,^^25^ The activity indices contain the following parameters: endocapillary hypercellularity, cellular crescents, karyorrhexis/fibrinoid necrosis, subendothelial hyaline deposits, interstitial inflammation, and leucocyte infiltration, whereas the chronicity indices include glomerular sclerosis, fibrous crescents, tubular atrophy, and interstitial fibrosis.

### Direct immunofluorescence examination

Direct immunofluorescence for immunoglobulin G (IgG), immunoglobulin A (IgA), immunoglobulin M (IgM), C3, C1q, and fibrin deposits were semi-quantitatively graded from 0 to 4 according to the intensity of fluorescence.

### Electron microscopy examination

Renal biopsy specimens were fixed in 2.5% paraformaldehyde for electron microscopy. After embedded in epon, ultrathin sections were mounted on metal grids and stained with uranyl acetate before viewed in a transmission electron microscope (JEM-1230; JEOL, Tokyo, Japan).

### Detection of serum PTX3 levels using ELISA

Serum levels of PTX3 were measured using a sandwich enzyme-linked immunosorbent assay (ELISA) based on the PTX3-specific monoclonal antibody MNB4 (Santa Cruz Technology, Santa Cruz, CA) and on biotinylated rabbit PTX3-specific polyclonal IgG (donated by Humanitas Mirasole s.p.a.).^16^ In brief, ELISA plates (Costar, Corning, NY) were coated with 2 μg/ml of monoclonal mouse anti-PTX3 antibody MNB4 diluted in bicarbonate buffer and were incubated overnight at 4 °C. The plates were washed three times with PBS with 0.1% Tween 20. After blocked with 1% bovine serum albumin (BSA) for 1 h at 37 °C, either recombinant human PTX3 standard or serum samples diluted 1:20 in PBS supplemented with 1% BSA and 10 mmol/L EDTA were added and incubated for 1 h at 37 °C. After three washes, biotinylated polyclonal rabbit anti-PTX3 antibodies diluted to 1:2000 was added for 1 h at 37 °C. Then, horseradish peroxidase (HRP)-conjugated streptavidin (Sigma, San Francisco, CA) diluted to 1:2000 was added. The reaction was developed with 3, 30, 5, 50-tetramethylbenzidine (TMB) liquid substrate system and was stopped with 1 M H_2_SO_4_. The results were recorded as the net optical absorbance at 450 nm and 570 nm in an ELISA reader (Bio-Rad 550, Hercules, CA). This assay was highly sensitive and specific, and no cross-reactions were observed with other pentraxins.^26^

Serial concentrations of commercial recombinant human PTX3 standard from 0 to 100 ng/ml were used to develop standard curve. The interassay and intraassay variances were 1.78% and 4.79% in multiple repeats, which proved that the method was accurate and reliable.

### Detection of serum anti-PTX3 auto-antibodies by ELISA

According to the previous study,^17^ human PTX3 (donated by Humanitas Mirasole s.p.a.), diluted to 5 μg/ml with phosphate buffered saline (PBS), was coated onto the wells of one half of a polystyrene microtiter plate (Costar, Corning, NY) and kept overnight at 4 °C. The wells in the other half were coated with phosphate buffered saline (PBS) alone to act as antigen-free wells. The plates were blocked with 1% bovine serum albumin (BSA) for 1 h at 37°C. Serum samples diluted 1:50 in PBS supplemented with 1% BSA and 10 mmol/L EDTA were added and incubated for 1 h at 37 °C. The plates were incubated with alkaline phosphatase-conjugated goat anti-human IgG (Sigma, St Louis, MO) for 1 h at 37 °C. Then the *p*-nitrophenyl phosphate (pNPP, 1 mg/ml; Sigma, St Louis, MO) was added and optical density (OD) was measured at 405 nm. The plates were washed three times after each step with PBS with 0.05% Tween-20. The cutoff value of anti-PTX3 auto-antibodies was set as the mean +2 SD of healthy blood donors.

### Statistical analysis

Statistical software SPSS 13.0 (SPSS, Chicago, IL) was performed for statistical analysis. Quantitative data were expressed as mean ± SD, or median (inter-quartile range) and categorical data were expressed as ratio. One-way analysis of variance was used for the same continuous data in different groups. Differences of quantitative variables between groups were assessed using the *t* test (for normally distributed data) or Mann–Whitney *U* test (for non-normally distributed data). The *χ*^2^ test was performed for compare categorical parameters between groups. The Pearson correlation and Spearman’s rank test were used for correlation analysis. Renal survival rates were assessed by Kaplan–Meyer method using log-rank test for comparison. All *p* values below 0.05 were considered statistically significant.

## Results

### General data of patients and controls

The general data of the patients with lupus nephritis are shown in Table 1. Gender and age were comparable between the lupus nephritis group (male/female: 33/163, 33.8 ± 11.4 years), SLE without renal involvement group (male/female: 17/113, 30.9 ± 13.7 years), and normal controls (male/female: 15/85, 34.4 ± 8.2 years) (*P* = .650, *p* = .514, respectively).

**Table 1.** General data of lupus nephritis patients.

Clinical evaluation	
Gender (male/female)	33/163
Age (mean ± SD) (years)	33.8 ± 11.4
Duration of follow-up (median and inter-quartile range) (months)	48; 12–84
SLEDAI (mean ± SD)	17.9 ± 6.0
Anemia no. (%)	114 (58.8)
Thrombocytopenia no. (%)	55 (28.4)
Leukocytopenia no. (%)	87 (44.8)
Hematuria no. (%)	151 (78.2)
Leukocyturia (non-infection) no. (%)	104 (53.6)
Acute renal failure no. (%)	39 (29.5)
Laboratory assessment	
Hemoglobin (mean ± SD) (g/l)	103.7 ± 26.3
Urine protein(median; inter-quartile range) (g/24 h)	4.33; 2.19–7.26
Serum creatinine (median; inter-quartile range) (μmol/l)	84; 68–138.3
Antinuclear antibody (+) no. (%)	192 (99.0)
Anti-dsDNA antibodies (+) no. (%)	136 (70.1)
Anti-cardiolipin antibodies (+) no. (%)	19 (15.4)
Anti-SSA antibodies (%) (+) no. (%)	87 (44.85)
Anti-SSB antibodies (%) (+) no. (%)	19 (9.8)
C3 (mean ± SD) (g/l)	0.48 ± 0.24
Pathological parameters	
Endocapillary hypercellularity (median; inter-quartile range)	3; 1–3
Cellular crescents (median; inter-quartile range)	0; 0–2
Karyorrhexis/fibrinoid necrosis (median; inter-quartile range)	0; 0–2
Subendothelial hyaline deposits (median; inter-quartile range)	1; 0–2
Interstitial inflammatory cell infiltration (median; inter-quartile range)	1; 1–1
Glomerular leukocyte infiltration (median; inter-quartile range)	1; 0–1
Activity indices (AIs) score(median; inter-quartile range)	8; 4–11
Glomerular sclerosis (median; inter-quartile range)	0; 0–1
Fibrous crescents (median; inter-quartile range)	0; 0–0
Tubular atrophy (median; inter-quartile range)	1; 1–1
Interstitial fibrosis (median; inter-quartile range)	1; 1–1
Chronicity indices (CIs) score (median; inter-quartile range)	2; 2–4

SD: standard deviation; SLEDAI: Systemic Lupus Erythematosus Disease Activity Index; dsDNA: double-stranded DNA; SSA: Sjögren’s syndrome A antigen; SSB: Sjögren’s syndrome B antigen.

**Table 2.** Comparisons of clinical, laboratory, and pathological data between patients with and without anti-PTX3 auto-antibodies in lupus nephritis.

	Anti-PTX3 antibodies (+),*n* = 38	Anti-PTX3 antibodies (−),*n* = 158	*p* Value
Clinical evaluation			
Gender (male %)	12 (31.6)	21 (13.3)	.01
Age (mean ± SD) (years)	33.42 ± 13.57	33.92 ± 10.89	.83
SLEDAI (mean ± SD)	19.41 ± 5.87	17.48 ± 5.98	.08
Anemia no. (%)	23 (60.5)	91 (58.3)	.81
Thrombocytopenia no. (%)	8 (21.1)	47 (30.1)	.27
Leukocytopenia no. (%)	19 (50.0)	68 (43.6)	.48
Hematuria no. (%)	33 (86.8)	118 (76.1)	.15
Leukocyturia (non-infection) no. (%)	25 (65.8)	79 (50.6)	.09
Acute renal failure no. (%)	9 (32.1)	30 (28.8)	.73
Laboratory assessment			
Hemoglobin (mean ± SD) (g/l)	98.58 ± 30.92	104.93 ± 25.00	.18
Serum creatinine (median; inter-quartile range) (μmol/l)	87.0; 74.75–143.50	82.5; 67.25–137.05	.27
Anti-dsDNA antibodies (+) no. (%)	29 (76.3)	107 (68.6)	.35
Anti-cardiolipin antibodies (+) no. (%)	5 (23.8)	15 (14.6)	.47
Pathological parameters			
Activity indices score (median; inter-quartile range)	9; 6.75–11.25	8; 4–11	.13
Chronicity indices score (median; inter-quartile range)	3; 2–3	2; 2–4	.70

SD: standard deviation; SLEDAI: Systemic Lupus Erythematosus Disease Activity Index; dsDNA: double-stranded DNA.

**Table 3.** Comparison of clinical, laboratory, pathological data, and scores of renal vascular lesions between different groups.

	Group 1, *n* = 54	Group 2, *n* = 11	*p* Value
Clinical evaluation			
Gender (male %)	11.1 (6/54)	18.2 (2/11)	.88
Age (mean ± SD) (years)	33.35 ± 10.38	34.00 ± 16.02	.90
SLEDAI (mean ± SD)	19.38 ± 7.04	19.91 ± 5.20	.78
Anemia no. (%)	29 (55.8)	6 (54.5)	.94
Thrombocytopenia no. (%)	18 (34.6)	4 (36.4)	.91
Leukocytopenia no. (%)	19 (36.5)	5 (45.5)	.58
Hematuria no. (%)	42 (82.4)	10 (90.9)	.48
Leukocyturia (noninfection) no. (%)	28 (53.8)	5 (45.5)	.61
Acute renal failure no. (%)	19 (48.7)	1 (14.3)	.12
Laboratory assessment			
Hemoglobin (mean ± SD) (g/l)	103.67 ± 21.83	102.55 ± 34.48	.89
Urine protein (median; inter-quartile range) (g/24 h)	4.79; 3.39–8.28	3.95; 1.78–7.0	.03
Serum creatinine (mean ± SD) (μmol/l)	168.84 ± 153.63	101.44 ± 47.36	.01
Anti-dsDNA antibodies (+) no. (%)	36 (69.2)	9 (81.8)	.40
Anti-cardiolipin antibodies (+) no. (%)	11 (31.4)	0 (0.0)	.35
Pathological parameters			
Activity indices score (median; inter-quartile range)	9; 5.5–12	9; 7–11	.66
Chronicity indices score (median; inter-quartile range)	2; 2–4	2; 2–3	.49
Thrombotic microangiopathy no. (%)	14/41 (34.1)	0/9 (0.0)	.04

SD: standard deviation; SLEDAI: Systemic Lupus Erythematosus Disease Activity Index; dsDNA: double-stranded DNA.

Patients in group 1 were without anti-PTX3 auto-antibodies and with higher serum PTX3 levels (≥3.207 ng/ml); patients in group 2 were with anti-PTX3 auto-antibodies and with lower PTX3 levels (<3.207 ng/ml).

### Serum PTX3 levels in patients and controls

The average level of serum PTX3 in active lupus nephritis patients was significantly higher than that in SLE without renal involvement (3.207 (0.399–56.376) ng/ml versus 1.065 (0.040–9.098) ng/ml, *p* < .001) and normal controls (3.207 (0.399–56.376) ng/ml versus 0.993 (0.007–2.655) ng/ml, *p* < .001). No difference was found in the average serum PTX3 levels between SLE without renal involvement and normal controls (*p* = .065).

### The prevalence of serum anti-PTX3auto-antibodies in patients and controls

**Figure 1.**
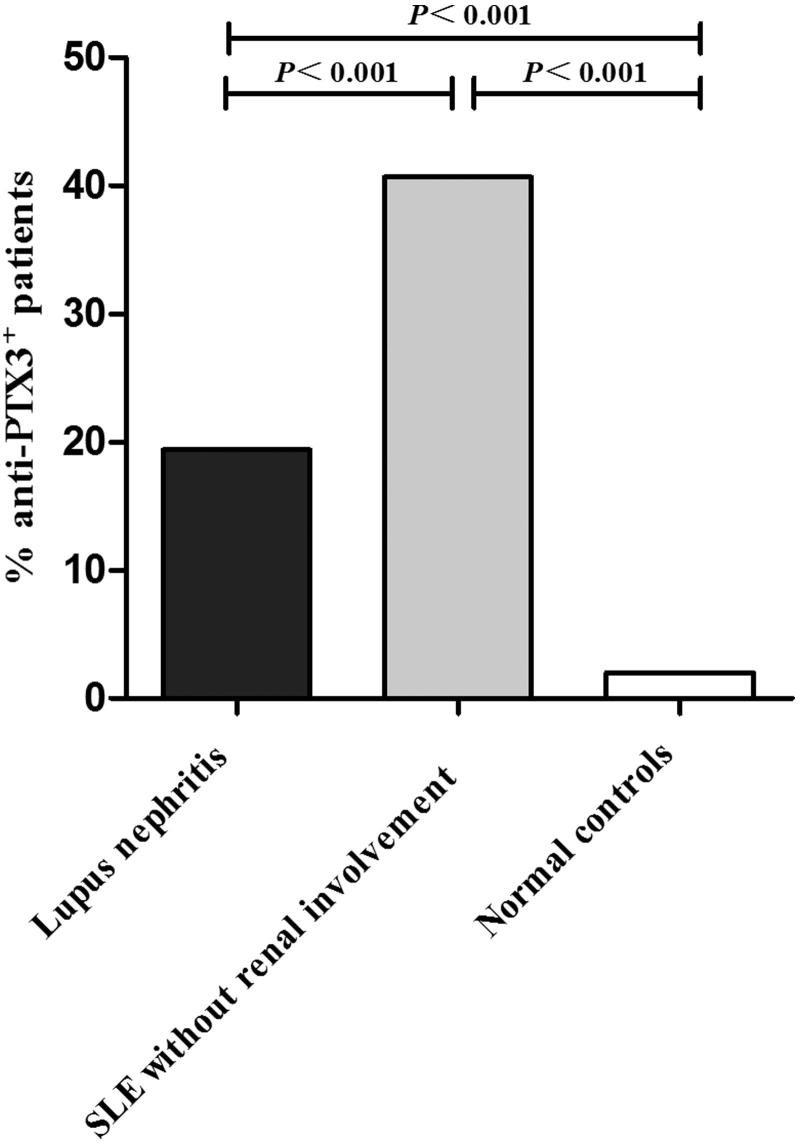
The prevalence of anti-PTX3 auto-antibodies in lupus patients and normal controls.

The positive ratio of anti-PTX3 auto-antibodies in SLE without the renal involvement group was significantly higher than that in the lupus nephritis group (40.7% (61/150) versus 19.4% (38/196), *p* < .001) and healthy controls (40.7% (61/150) versus 2% (2/100), *p* < .001). The positive ratio of anti-PTX3 auto-antibodies in lupus nephritis was significantly higher than that in normal controls (19.4% (38/196) versus 2% (2/100), *p* < .001) (Figure 1).

### Comparisons of clinicopathological data between patients with and without anti-PTX3auto-antibodies in lupus nephritis (Table 2)

Male patients with lupus nephritis was more likely to have anti-PTX3 auto-antibodies than females (31.6% (12/38) versus 13.3% (21/158), *p* = .01). There was no significant difference of age between the patients with and without anti-PTX3 antibodies (33.92 ± 10.89 versus 33.42 ± 13.57, *p* = .83).

The serum levels of anti-PTX3 auto-antibodies were negatively correlated with the amount of proteinuria in lupus nephritis patients (*r*= −.143, *p* = .47). No differences in other clinicopathological features were found between patients with or without anti-PTX3 auto-antibodies in lupus nephritis. There were no significant correlations between serum PTX3 and anti-PTX3 auto-antibodies levels in lupus nephritis patients (*r* = .074, *p* = .374).

**Figure 2.**
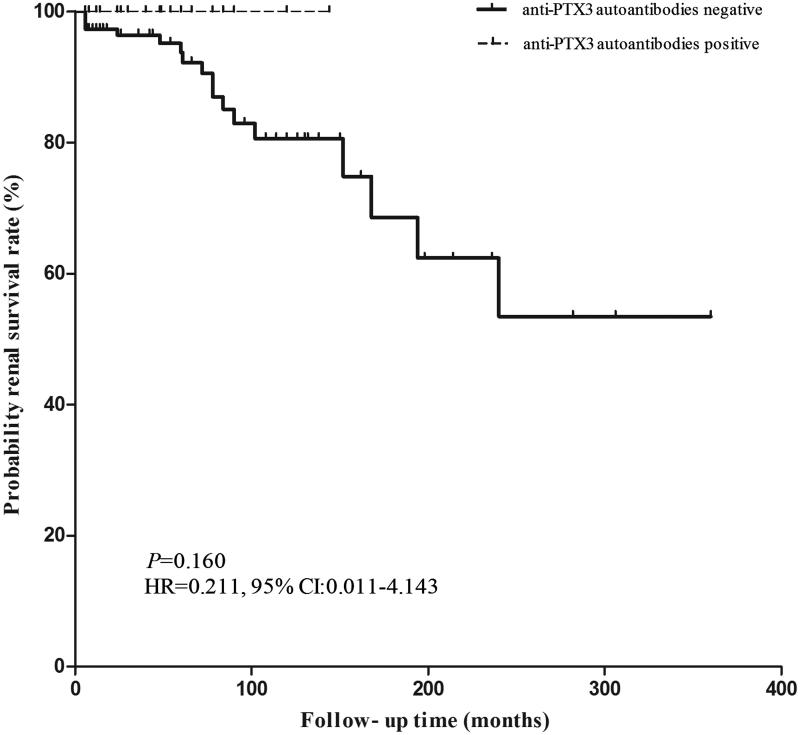
Comparison of renal outcomes between patients with and without anti-PTX3 auto-antibodies.

The patients with lupus nephritis were followed up with a duration of 48 (range 6–360) months. There was no significant difference of renal survival (including ESRD or doubling of serum creatinine levels) rate between patients with and without anti-PTX3 auto-antibodies (*p* = .160, HR =0.211, 95% CI: 0.011–4.143) (Figure 2).

### Comparisons of clinicopathological data between patients with anti-PTX3 auto-antibodies and low levels of PTX3 and patients without anti-PTX3 auto-antibodies and high levels of PTX3 (Table 3)

As serum PTX3 levels was closely associated with disease activity and severity of lupus nephritis in previous studies,^16^^,^^18^^,^^27^ and PTX3 deposition and renal fibrosis were significantly lower in anti-PTX3 auto-antibodies positive patients compared with those negative, confirmed by experimental animal model,^17–19^ we speculated that anti-PTX3 auto-antibodies and PTX3 might exert opposite effect under pathological conditions. Thus, the lupus nephritis patients in our study were further divided into two groups according to their serum anti-PTX3 auto-antibodies and PTX3 levels: patients in group 1 were anti-PTX3 auto-antibodies negative and with higher serum PTX3 levels (≥3.207 ng/ml), while patients in group 2 were anti-PTX3 auto-antibodies positive and with lower PTX3 levels (<3.207 ng/ml).

Patients in group 1 presented with significantly higher levels of proteinuria and serum creatinine compared with those from group 2 (4.79 (3.39–8.28) versus 3.95 (1.78–7.0) g/24 h, *p* = .03; 168.84 ± 153.63 versus 101.44 ± 47.36 μmol/l, *p* = .01, respectively). Moreover, the prevalence of renal pathological thrombotic microangiopathy was significantly higher in group 1 compared with that in group 2 (34.1% (14/41) versus 0% (0/9), *p* = .04).

## Discussion

In the current study, we confirmed that anti-PTX3 auto-antibodies were less prevalent in active lupus nephritis patients compared with SLE without renal involvement, which was consistent with the previous study.^18^ Although it did not reach statistical significance, there was a trend that the patients with anti-PTX3 auto-antibodies negative had lower cumulative renal survival rate in comparison with those positive during follow-up time. Recent study with the murine models of lupus also demonstrated that immunization with PTX3 and subsequent development of anti-PTX3 auto-antibodies significantly delayed occurrence of nephritogenic antibodies, decreased proteinuria, and increased survival.^19^ More interestingly, we found that the positive ratio of anti-PTX3 auto-antibodies was higher in male lupus nephritis than females. It might be attributed to the following reasons: first, from the pathogenic view of SLE, hydroxylation of estrone and androgens dysfunction might affect the immune system of patients.^28–30^ and male lupus patients were more likely to have auto-antibodies, like anti-dsDNA antibodies, anti-Sm antibodies, anti-cardiolipin antibodies, etc. as shown in some studies.^31^^,^^32^ Second, several studies showed that the long-term survival and renal outcomes were better in male lupus nephritis patients compared with females^33^^,^^34^ which might be in consistence with our speculation that anti-PTX3 auto-antibodies could be protective.

Previous studies, including ours, indicated that serum PTX3 level was closely associated with disease activity and severity of lupus nephritis.^16^^,^^18^^,^^27^ Moreover, PTX3 deposition and renal fibrosis were significantly lower in anti-PTX3 auto-antibodies positive patients compared with those negative, which was consistent with the results of experimental animal model,^17–19^ suggesting anti-PTX3 auto-antibodies and PTX3 might exert opposite effect under pathological conditions. Thus, lupus nephritis patients in our study were divided into two groups according to their serum anti-PTX3 auto-antibodies and PTX3 levels. Interestingly, we found that the group with anti-PTX3 auto-antibodies negative and higher levels of serum PTX3 presented with more severe renal injury, like higher proteinuria amount, higher serum creatinine value and higher ratio of TMA change than patients with anti-PTX3 auto-antibodies positive and lower levels of serum PTX3, which suggested that the balance between serum PTX3 and anti-PTX3 auto-antibodies levels might influence the progression or attenuation of the nephritic process *in situ*. It indicated that the combination of serum PTX3 level and anti-PTX3 auto-antibodies could predict lupus nephritis activity better, not the whole SLE disease activity as the SLEDAI scores were comparable between the two groups. It might also highlight the protective effect of anti-PTX3 auto-antibodies indirectly in human lupus nephritis.

A recent study from Bassi et al. showed that the prevalence of both anti-dsDNA and anti-C1q antibodies was significantly higher in SLE patients with renal involvement than those without lupus nephritis, whereas the ratio of anti-PTX3 auto-antibodies positive was opposite.^18^ In addition, Gatto et al. found that anti-dsDNA and anti-C1q antibodies were at lower titers in anti-PTX3 auto-antibodies positive mice than control groups. Moreover, the occurrence of anti-dsDNA and anti-C1q antibodies in PTX3 immunized mice was significantly delayed, and they both started to increase when anti-PTX3 auto-antibodies was fading, proposing that anti-PTX3 auto-antibodies might inhibit secretion of nephritogenic antibodies in lupus nephritis.^19^

The role of anti-PTX3 auto-antibodies in the pathogenesis of lupus nephritis remained unclear. The study from our lab indicated that PTX3 might be expressed mainly by fibroblast in tubulo-interstitial areas of lupus nephritis patients,^16^ which led to the hypothesis that the pro-inflammatory and pro-fibrotic effects of PTX3 was prevailing under the micro-environment of lupus nephritis, and highlight the associations between PTX3 and interstitial inflammation and fibrosis *in situ*. Meanwhile, Bassi et al. recently found that the PTX3 level and the degree of renal fibrosis were both higher in patients without anti-PTX3 auto-antibodies than those positive in lupus nephritis.^18^ It could be speculated that anti-PTX3 auto-antibodies might prevent the deposition of PTX3 in kidneys, thus hindering lupus nephritis progress, although the real bio-functions of anti-PTX3 auto-antibodies was still not clear *in vivo*. Interestingly, recent studies indicated that IgM anti-dsDNA antibodies might down-regulate autoreactive B cells and decrease the production of pathogenic IgG anti-dsDNA antibodies and hinder formation of IgG containing immune complexes against lupus nephritis.^35^ Thus, it could be inferred that anti-PTX3 auto-antibodies might similarly disturb renal intrinsic cells and decrease the secretion of PTX3, which needs further exploration. Moreover, several studies have shown that PTX3 might exert a pathogenic role by inhibiting the removal of apoptotic materials, generating abundant auto-antibodies, amplifying the immune response, and enhancing complement activation.^36–39^ Membrane-bound PTX3 on apoptotic cells is likely to be bound by C1q, thus promoting excessive activation of the classical complement pathway.^40^ Interestingly, studies *in vitro* found that the binding of C1q to immune complexes formed by anti-PTX3 antibodies and solid-phase immobilized PTX3 could be affected by the presence of anti-PTX3 auto-antibodies. It is very likely that the presence of anti-PTX3 might also be an interference of complement activation.^19^

Our work has some limitations. First, it was a retrospective study with small sample size, which might limit its significance. Second, the mechanic investigations by which anti-PTX3 auto-antibodies could modify disease course, especially its immunologic characteristics in lupus nephritis, were needed in the future.

In conclusion, anti-PTX3 auto-antibodies were less prevalent in active lupus nephritis patients compared with SLE without renal involvement and associated with less severe renal damage, especially with the combined evaluation of serum PTX3 levels.
